# Emergency department encounters for opioid abuse, adverse events, poisoning, and dependence among members of a community-based health insurance plan—Central Texas, 2016–2018

**DOI:** 10.1186/s12889-019-7394-9

**Published:** 2019-08-13

**Authors:** John R. Litaker, Naomi Tamez, Wesley Durkalski, Richard Taylor

**Affiliations:** 1 The Litaker Group, LLC, P. O. Box 160505, Austin, TX 78716 USA; 2 Sendero Health Plans, 2028 E Ben White Blvd #400, Austin, TX 78741 USA; 3 University of Texas at Austin, 200 W 24th St, Stop A2700, Austin, TX 78712-1247 USA

**Keywords:** Opioids, Health maintenance organization, Emergency department encounters, Affordable Care Act, Sendero Health Plans

## Abstract

**Background:**

The United States appears to be in the midst of an opioid epidemic. National data indicate a rise in emergency department visits for opioid-related causes over the past decade. This data, while important in helping to explain the magnitude of the epidemic nationally offers only a glimpse of what can be expected to occur locally. The objective of this secondary data analysis was to describe the impact that opioid abuse, adverse events, poisoning, and dependence have on emergency department utilization for individuals who purchased health insurance under the Affordable Care Act in Central Texas from a community-based health maintenance organization.

**Methods:**

Individuals who purchased health insurance from Sendero Health Plans in calendar years 2016, 2017, and 2018 were eligible for participation if they had *both* an emergency department encounter and an opioid-related ICD-10-CM diagnosis. Eligible individuals were assessed to determine if they were dispensed an opioid agonist or opioid antagonist prescription during the year of their emergency department encounter. Sendero medical claims data for calendar years 2016, 2017, and 2018 were used to calculate both the incidence and ratio of emergency department visits per 100,000-person Sendero member population. Sendero data were compared to available national data estimates.

**Results:**

A total of 55 individuals had an emergency department encounter with a primary or secondary opioid-related diagnosis from January 1, 2016 through December 31, 2018. These 55 individuals had 69 unique emergency department encounters during this time period. The incidence of new claims per 100,000-member Sendero population was 67.1, 64.5, and 62.6 in 2016, 2017, and 2018 respectively. The ratio of unique emergency department encounters per 100,000-member Sendero population was 95.9, 82.6, and 66.5 in 2016, 2017, and 2018 respectively.

**Conclusion:**

Health insurance claims data from a community-based health plan can be used as a source of local information by policy makers and officials as they seek to address the impact of opioid abuse, adverse events, poisoning, and dependence in Central Texas as national data may not represent the local impact of this epidemic.

## Background

The United States appears to be in the midst of an opioid epidemic. Nationally, opioid-related mortality—including deaths from synthetic opioids, commonly prescribed opioids, and heroin—increased from 3.0 per 100,000 persons in 2000 to 14.9 per 100,000 persons in 2017 [1]. An early alarm was raised on the impact of opioid-related poisonings in 2006 in the United States with data showing an increase in US hospital admissions by 65% from 1999 to 2006 due to poisoning by prescription opioids, sedatives, and tranquilizers [2]. Additional research from 2005 to 2014 showed an increase in opioid prescriptions, opioid poisoning, and opioid-related utilization of inpatient services and emergency department resources [3–6]. Analyses from the Agency for Healthcare Research and Quality (AHRQ) using data from the Healthcare Cost and Utilization Project (HCUP) showed cumulative increases of 64.1% and 99.4% among inpatient and emergency department visits, respectively, due to opioid-related diagnoses from 2005 to 2014 [7].

In October 2017 the US Department of Health and Human Services (HHS) declared the opioid crisis in the United States to be a public health emergency [8]. With that declaration, HHS released a five-point strategy to combat the opioid crisis with a focus on access to treatment, improved data, better pain management, increased use of overdose-reversing medications, and better research on pain and addiction [9]. Implicit in this five-point strategy is the need for data at the local level to address interventions based on local conditions.

Within Central Texas, a review of drug overdose and opioid use in Travis County, Texas showed an increase in overdose deaths from 4.0 per 100,000 population in 2006 to 7.5 per 100,000 in 2016 [10]. During this time, 590 deaths were attributed to opioids, with heroin identified in 262 persons (44.4%) [10]. In May 2018 the Austin, Texas City Council resolved to address the public health and public safety issues associated with opioid use and overdose [11]. Among the items in this resolution was a call to action to improve epidemiological surveillance and monitoring related to opioid use [11].

In July 2018 the local health department, Austin Public Health, convened a stakeholder group of community partners and organizations to discuss this resolution and to identify opportunities to strengthen collaboration to identify and use locally available data to help make evidence-based decisions on how to respond to opioid use and abuse in Central Texas. A second meeting was held in January 2019. Sendero Health Plans, Inc. (Sendero), a community-based health maintenance organization in Austin, Texas providing health insurance coverage under the Affordable Care Act (ACA) was invited to participate in these discussions.

The Sendero perspective on this issue is important for three reasons: (1) Sendero is a community-based, taxpayer supported health plan that recognizes the importance of providing evidence-based data to assist decision making on matters of community importance; (2) Sendero is a provider of health insurance to individuals under the ACA and is able to provide aggregate data and analytical information on how opioid use is affecting a portion of the population in Central Texas; and (3) some Sendero members include local musicians and others who, according to the City of Austin resolution, may have been impacted by opioid use.

The objective of this secondary data analysis is to describe the impact that opioid abuse, adverse events, poisoning, and dependence have on emergency department utilization for Sendero members who purchased health insurance as part of the ACA. This present study also seeks to provide local data to help local decision-makers address this issue. Finally, this study seeks to contribute information in support of the improved data component outlined in the US Department of Health and Human Services opioid response strategy.

## Methods

The study population includes individuals who live in an eight-county area of Central Texas and who purchased health insurance on the federal marketplace under the ACA from Sendero during 2016, 2017, and 2018. Data were obtained from Sendero medical claims, prescription, and membership data. Individuals who purchase an ACA health insurance plan from Sendero often do not have access to health insurance through their employer, many are self-employed, and some may receive premium subsidy assistance in order to purchase health insurance.

Calendar year 2016 was selected as the initial time period for this study because it is the first full year in which ICD-10-CM (International Classification of Diseases, Tenth Revision) codes were used. This allowed for comparison to future years without need to make adjustments for the changeover from ICD-9 (International Classification of Diseases, Ninth Revision) to ICD-10. Calendar year 2018 was chosen as the end time period because it is the most recent calendar year in which complete data were available.

The study population was identified as follows:

1. Individuals with emergency department encounters were identified on the basis of a Current Procedural Terminology (CPT) code for an emergency department encounter and either a primary or secondary opioid-related ICD-10-CM code. The five CPT codes used to identify an emergency department encounter were 99281, 99282, 99283, 99284, and 99285. The ICD-10-CM codes used to determine an opioid-related diagnosis were based on AHRQ criteria, details of which are described elsewhere [7, 12].
2. Data were then stratified to identify claims processed using the US Centers for Medicare and Medicaid Uniform Billing Form number 04 (UB-04). The UB-04 confirms that an encounter took place at a physical location.
3. Further stratification separated claims into two groups: (1) unique *encounters*; and (2) unique *members*. A unique *encounter* is defined as a single emergency department encounter that included a service “from” date and service “to” date for emergency department care that does not include an inpatient admission to the hospital. A member may have more than one unique encounter during each calendar year. A unique *member* is described as a member who had one or more emergency department encounter(s) that included a service “from” date and service “to” date from the emergency department during the calendar year that does not include an inpatient admission to the hospital.

Opioid-related ICD-10-CM codes were assigned to one of four categories to reflect the type of opioid encounter based on AHRQ [12] criteria: (1) opioid abuse; (2) adverse effects of opioids; (3) opioid dependence and unspecified use; and (4) opioid poisoning. Details of the AHRQ methodology and ICD-10-CM codes assigned to each of the four groups are available elsewhere [12].

Individuals who had an opioid-related emergency department encounter were further assessed to determine if they had an opioid-related prescription during the calendar year of their emergency department encounter. Opioid-related prescriptions were stratified as an agonist or antagonist. For this study we recognize that some medications include both partial agonist (e.g., buprenorphine) and antagonist (e.g., naloxone) properties; we refer to medications containing these two active ingredients as antagonists since the primary indication for buprenorphine and naloxone combination drugs is to assist individuals with symptoms of withdrawal when ceasing opioid use. Prescription claim data for 2016 through 2018 were matched against National Drug Code (NDC) data for opioid-related medications prepared by the US Centers for Disease Control and Prevention (September 2017; NDC codes available upon request). Pharmacy claims data provided information on both quantity of medication ordered, and quantity dispensed; only prescriptions that indicated a medication was actually dispensed were included in the analysis.

Data were analyzed using IBM® SPSS® Statistics version 26 and Microsoft® Excel for Mac version 16.21.1. The following demographic and clinical characteristics of opioid-related encounters were analyzed: age, sex, primary opioid-related ICD-10-CM diagnosis code, secondary ICD-10-CM diagnosis code, and prescription type. The primary diagnosis code is that ICD-10-CM code determined to be “chiefly responsible for the patient’s [encounter]” while secondary diagnosis codes “are concomitant conditions that coexist at the time of encounter.” [7] The unit of analysis is either the unique *encounter* or unique *member* who presented at the emergency department with an opioid-related primary or secondary ICD-10-CM code.

Reported ratio and incidence data are restricted to Sendero member population data for the calendar year in which an emergency department visit occurred. Ratios for each calendar year represent the total number of claims associated with all unique encounters at the emergency department per 100,000-member Sendero population. Additionally, the reported incidence for each calendar year represents the total number of first-time claims of unique member encounters at the emergency department per 100,000-member Sendero population.

## Results

### Emergency department visit for opioid-related ICD-10 diagnoses

For 2016 the member population size was 20,854 followed by 38,746 in 2017 and 25,568 in 2018. A search of emergency department encounters for opioid-related ICD-10-CM codes for the period of January 1, 2016 through December 31, 2018 identified 55 individuals with an emergency department encounter of a primary or secondary opioid-related diagnosis. Of the 55 *individuals* with a claim during the 3 years, 14 (25.5%) had an emergency department encounter in 2016, 25 (45.5%) had an emergency department encounter in 2017, and 16 (29.1%) had an emergency department encounter in 2018 (Table 1). One member had an opioid-related emergency department encounter in calendar years 2016 and 2017. A different member had an opioid-related emergency department encounter in calendars year 2017 and 2018.

**Table 1:** Number of individuals with opioid-related claim and number of opioid-related claims at an emergency department in calendar years 2016–2018

	2016	2017	2018	Total
Number of individuals with an opioid-related emergency department encounter	14	25	16	55
Number of claims for an opioid-related emergency department encounter	20	32	17	69
Sendero member population	20,854	38,746	25,568	N/A

*N/A* Not applicable

A total of 69 encounters for an opioid-related primary or secondary diagnosis were made in the period of January 1, 2016 through December 31, 2018. Of these 69 total unique emergency department encounters for Sendero members, 20 (29.0%) occurred in calendar year 2016, 32 (46.4%) occurred in calendar year 2017, and 17 (24.6%) occurred in calendar year 2018.

Table 2 provides a summary of claim incidence and claim ratios for the three-year study period. The incidence of new member claims per 100,000-member Sendero population is 67.1, 64.5, and 62.6 in calendar years 2016, 2017, and 2018 respectively. The ratio of claims representing the total of all encounters per 100,000-member Sendero population is 95.9, 82.6, and 66.5 for 2016, 2017, and 2018 respectively.

**Table 2:** Incidence of first time claims and ratio of encounters for opioid-related emergency department visits from 2016–2018

	2016	2017	2018
Incidence of First-Time Claims for an Opioid-Related ED Encounter per 100,000 Member Population	67.1	64.5	62.6
Ratio of Emergency Department Encounters for an Opioid-Related ED Encounter per 100,000 Member Population	95.9	82.6	66.5

*ED* Emergency Department

In 2016 the mean age of individuals with a primary or secondary opioid-related ICD-10-CM diagnosis code was 34.1 ± 11.7 years (range, 19–54 years). In 2017 the mean age was 35.0 ± 11.2 years (range, 19–56 years) and in 2018 the mean age was 36.3 ± 13.0 years (range, < 18–64 years). During the three-year study period, the mean age tended to trend slightly higher and tended to have a slightly wider range. (See Table 3.)

**Table 3:** Sex and age summary of unique members who had an emergency department encounter in calendar years 2016–2018 that included an opioid-related primary or secondary ICD-10-CM diagnosis

Variable	2016	2017	2018
Sex	n (%)	n (%)	n (%)
Female	11 (55.0%)	13 (40.6%)	11 (64.7%)
Male	9 (45.0%)	19 (59.4%)	6 (35.3%)
Age in Years			
Mean (± SD)	34.1 (±11.7)	35.0 (±11.2)	36.3 (±13.0)
Median	32.0	32.0	35.0
Range	19–54	19–56	< 18–64

*SD* Standard Deviation

Females represented the majority of the total number of opioid-related emergency department encounters for both 2016 and 2018. Of the 20 unique encounters during 2016 males had nine encounters (45.0%) and females had 11 encounters (55.0%). Of the 32 unique encounters during 2017, males had 19 encounters (59.4%) and females had 13 encounters (40.6%). Of the 17 unique encounters during 2018, males had six encounters (35.3%) and females had 11 encounters (64.7%). (See Table 3.)

Opioid-related ICD-10-CM diagnosis codes were assigned to one of four groups based on a stratification protocol reported by AHRQ. ^17^ The four groups include opioid abuse, adverse effects of opioids, opioid dependence and unspecified use, and opioid poisoning (see Table 4). Details of ICD-10-CM diagnosis code assignments to one of the four groups is available elsewhere [12].

**Table 4:** ICD-10-CM diagnosis codes by opioid-related ICD-10-CM category for calendar years 2016–2018 for number of claims for an opioid-related emergency department encounter

Opioid-Related ICD-10-CM Category	2016	2017	2018	Total
Opioid abuse	10 (50.0%)	9 (28.1%)	4 (23.5%)	23 (33.5%)
Adverse effects of opioids	0 (0.0%)	4 (12.5%)	1 (5.9%)	5 (7.2%)
Opioid dependencies and unspecified use	10 (50.0%)	16 (50.0%)	9 (52.9%)	35 (50.7%)
Opioid poisoning	0 (0.0%)	3 (9.4%)	3 (17.6%)	6 (8.7%)
Total	20 (100%)	32 (100%)	17 (100%)	69 (100%)

Table 5 summarizes the top three most frequently reported opioid-related ICD-10-CM codes for each year. ICD-10-CM codes were consistent across 2016 and 2017 with the three most frequently reported ICD-10-CM codes being F11.10 (opioid abuse, uncomplicated), followed by F11.20 (opioid dependence, uncomplicated), and F11.23 (opioid dependence, with withdrawal). In 2018 the most frequently reported code was F11.23 (opioid dependence, with withdrawal) followed by equally reported codes for F11.10 (opioid abuse, uncomplicated), and T40.1X1A (poisoning by other opioids, accidental).

**Table 5:** Most frequently reported opioid-related ICD-10-CM codes reported for an opioid-related emergency department encounter in calendar years 2016–2018 for number of claims for an opioid-related emergency department encounter

ICD-10-CM	Description	2016		2017		2018		Total	
		n	%	n	%	n	%	n	%
F11.10	Opioid abuse, uncomplicated	9	50.0	9	40.9	3	25.0	21	39.6
F11.20	Opioid dependence, uncomplicated	7	38.9	7	31.8	0	0.0	14	26.4
F11.23	Opioid dependence, with withdrawal	2	11.1	6	27.3	4	33.3	12	22.6
T40.1X1A	Poisoning by other opioids, accidental (unintentional, initial encounter)	0	0.0	0	0.0	3	25.0	3	5.7

### Pharmacy claim data for individuals who had an emergency department claim

Of the 55 *individuals* who had either a primary or secondary opioid-related diagnosis code for an emergency department encounter from 2016 through 2018, 38 (69.1%) individuals had 323 opioid-related prescriptions with 521 refills, for a total of 844 opioid-related prescriptions dispensed. Of the 844 opioid-related prescriptions 661 (78.3%) were for opioid agonists and 183 (21.7%) were for opioid antagonists. The top three agonists prescribed over the three-year time period were hydrocodone/APAP Tables 10–325 mg (*n* = 112, 16.9%), APAP/codeine tablets 300–30 mg (*n* = 106, 16.0%), and hydrocodone/APAP tablets 7.5–325 mg (*n* = 94, 14.2%). The three most prescribed antagonists during the three-year time period were Suboxone MIS 8–2 mg (*n* = 105, 57.4%), Bunavail MIS 4.2–0.7 mg (*n* = 45, 24.6%), and Suboxone MIS 4–1 mg (*n* = 13, 7.1%) all of which have buprenorphine and naloxone as active ingredients to prevent withdrawal.

## Discussion

Within the three-year study period, there was a decline from 67.1 to 62.6 in new incident cases of opioid-related emergency department encounters per 100,000-person Sendero member population. We do not know the reason for this decline, but it may reflect additional attention to this issue through health promotion and harm reduction initiatives at the local level or it may reflect changing demographics of the Sendero member population. Comparison data at the national level for new incident cases is not available. However, a recent assessment of racial, ethnic, and income disparities for opioid prescriptions in California indicates that exposure to opioids may be higher among individuals living in a majority-white area than in non-white areas [13]. While race or ethnicity questions are optional for ACA applicants, it is believed that Sendero’s membership reflects the Austin area distribution of race and ethnicity of 50% non-Hispanic white, 35% Latino, and 8% African American. Additional trend analysis across time was not performed due to violations of independence of observations.

One metric in which national level comparison data is available is the number of opioid-related emergency department encounters per 100,000 persons. Specifically, AHRQ analyzed data from the Nationwide Emergency Department Sample (NEDS) to arrive at a nationwide average of 177 encounters per 100,000 persons in 2014 [7]. (Calendar year 2014 data is the most recently available data.) Corresponding data from our study population show 95.9, 82.6, and 66.5 encounters per 100,000-member Sendero population in 2016, 2017, and 2018, respectively. We report our encounter data as a ratio, whereas AHRQ reports their encounter data as a rate. Comparing the local burden of opioid-related encounters in our population to the AHRQ national average shows a substantial difference in the number of emergency department encounters locally. In fact, our data are more in line with some of the lower reported state averages like Georgia and Kansas at 95.4 and 81.2 encounters per 100,000 population, respectively. Unfortunately, Texas is one of 20 states that does not report to (NEDS), which is the source data for the AHRQ research brief.

It should be noted that while AHRQ provides a source of comparison for encounters per 100,000 population, there are methodological concerns related to the AHRQ study. Specifically, the NEDS dataset used by AHRQ is based on cluster sample data from select hospitals in participating states. Because of this, the generalizability of NEDS data to create a state or national average is discouraged [14]. However, for our purposes, the AHRQ findings provide a source of comparison which, while methodologically challenged, is nevertheless a benchmark.

In reviewing prescription data as a post hoc analysis to the main study, 9 of 14 (64.3%) persons from the emergency department cohort in 2016, 20 of 25 (80.0%) persons in 2017, and 9 of 16 (56.3%) persons in 2018 were prescribed an opioid-related drug either before or after the emergency department encounter. When viewed by drug action (agonist or antagonist) the nine persons in 2016 were dispensed 61 agonist and 15 antagonist prescriptions. In 2017, the 20 persons were dispensed 423 agonist and 60 antagonist prescriptions. In 2018, the nine persons were dispensed 177 agonist and 108 antagonist prescriptions.

The prescription drug data provides an interesting glimpse into the role that ethical drugs play in opioid-related abuse, adverse events, poisoning, and dependence. While additional analysis is needed, a couple of points can be gleaned from the data presented here. Firstly, not everyone who has an opioid-related emergency department encounter has an opioid agonist prescription. Evidence from our members indicate that 31 of 55 (56.4%) persons with an opioid-related emergency department encounter were also dispensed an opioid agonist during the year of their emergency department encounter. This indicates that the other 43.6% of members who sought care at the emergency department for an opioid-related encounter were potentially using illegal opioids, agonist medications prescribed to someone else, or were obtaining agonist medications outside of the Sendero pharmaceutical benefits management plan. Regardless, these individuals are presenting for an opioid-related illness without evidence of an opioid agonist prescription. Secondly, our data indicated that 11 of 55 (20.0%) persons with an opioid-related emergency department encounter were also dispensed an opioid antagonist during the year of their emergency department encounter, presumably to lessen opioid withdrawal symptoms. This indicates that individuals who are trying to stop using opioids may relapse and thus require emergent or urgent care, or it may indicate they have had an adverse outcome with opioid use and have sought care at the emergency department, at which point they may have been prescribed an antagonist. Finally, among the populations represented in this study, a handful of individuals represent the vast majority of opioid agonists prescribed, with 7 of 55 Sendero members with opioid-related primary or secondary ICD-10-CM emergency department visit accounting for 322 of 661 (48.7%) agonist prescriptions. This represents an opportunity for Sendero to work with these members and their prescribing physician(s) to determine if these medications are appropriate.

In assessing data from our study, we reviewed a recent publication that analyzed opioid overdose data from Central Texas. This study reported lower opioid overdose deaths in Travis County, Texas than in the United States as a whole [10]. From the 11-year period of 2006–2018, the mortality rate of an opioid-related drug overdose was reported as 4.8 per 100,000 in Travis County, which is about half the rate reported nationally of 8.0 per 100,000 over the same time period [10]. When heroin and methadone are removed as a cause of drug overdose, semisynthetic opioids like hydrocodone and oxycodone had an overdose death rate of 1.8 per 100,000 (*n* = 205) and synthetic opioids like fentanyl and tramadol had an overdose death rate of 0.8 per 100,000 (*n* = 88) over the 11-year time period [10]. These findings, along with our findings, indicate a locally lower trend in Central Texas than nationally for opioid-related health outcomes and resource utilization.

The Sendero membership fluctuated during the three-year study period. Factors that influence membership include cost of monthly premium, annual deductible, type of benefits provided, the physician and hospital network, amount of any federal premium assistance or cost share, and comparison plan offerings by other health insurance companies. Individuals weigh these factors when making a decision to select the most appropriate health insurance product for themselves and / or their family. These factors may have influenced the findings of this study.

### Limitations

This study has several limitations. Firstly, this study is based on a small subset of the Central Texas population who have purchased health insurance from a community-based health plan. As such, this study does not, nor was it designed to, provide a comprehensive picture of the opioid epidemic in Central Texas. Secondly, this study only reviews medical claims for opioid-related emergency department encounters. Emergency department encounters are just a part of the overall opioid epidemic with other parts being emergency medical service calls that result in treatment but not transport, inpatient hospital admissions, and individuals who are able to manage their opioid use without the need to seek urgent or emergent medical assistance. We are not able to account for these individuals in our data. Thirdly, these data do not allow for long-term trend analysis and are limited to post ICD-10-CM data coding. Finally, while the number of persons with health insurance has increased since the introduction of the ACA, there remain people who are either uninsured or underinsured. The uninsured will not be represented in a health insurance claims database and the underinsured, while technically having health insurance coverage, might not seek medical care as they may find copays or cost sharing associated with seeking care unaffordable.

Future research on this topic is needed not only to identify additional data that can be used to support local decision making, but to better define the local burden of the epidemic. For example, additional data on opioid-related inpatient admissions, the use of naloxone in the community as a harm reduction activity, and a descriptive analysis of agonist and antagonist opioid prescription medications in the community is needed to help complete the local story of the opioid epidemic.

## Conclusion

National data has been used to describe the ongoing opioid epidemic across the United States. Such data is important in framing the national conversation and in initiating discussions at the local level. In Austin, Texas a call to action was issued by elected leaders to identify and use local data to assist policy makers and officials in making evidence-based decisions on how best to respond to opioid use and abuse in Central Texas. As a community health plan operated on behalf of Travis County taxpayers, Sendero answered this call to action with a three-year review of medical claims data and prescription drug data for opioid-related emergency department visits among its member population. We find that Sendero members have a lower incidence and ratio of opioid-related emergency department visits than comparable national studies.

We believe that in order to properly frame this conversation at the local level researchers, practitioners, and policy makers need to do a better job of defining the complexity of the opioid epidemic problem. There are many outlets, reports, and publications that indicate the nation is in the midst of an opioid crisis and in many cities across the country this may be the case. Yet, as we have seen with our study, findings within our popluation indicate a lower burden of impact than what is indicated by national data. Therefore, one could ask the question as to whether the national opioid crisis, as identified by the US Health and Human Services Department, is also a local crisis—at least in Central Texas—or if it is rather a local problem that has yet to achieve a crisis level designation? In order to frame this conversation properly—both nationally and locally—the right words must be used, and we must not just assume that national data is representative of local data until local data supports such a claim.

In closing, local data should be used whenever feasible to help inform local decision-making about the local impact of health issues. In fact, using local data to describe the local burden of disease can help provide the necessary local perspective on a national crisis. However, until such time that local data is routinely available, we will necessarily need to rely on national data as a guide—but only a guide—as to what we may expect to occur locally.

## Data Availability

The datasets generated and/or analyzed during the current study are not publicly available due HIPAA regulations but are available from the corresponding author on reasonable request.

## Abbreviations

ACA: Patient Protection and Affordable Care Act
AHRQ: Agency for Healthcare Quality and Research
CDC: US Centers for Disease Control and Prevention
CPT: Current Procedural Terminology
HCUP: Healthcare Cost and Utilization Project
HHS: Health and Human Services, US
HIPAA: Health Insurance Portability and Accountability Act
ICD-10-CM: International Classification of Disease, 10th Edition, Clinical Management
ICD-9-CM: International Classification of Disease, 9th Edition, Clinical Management
NDC: National Drug Code
NEDS: Nationwide Emergency Department Sample
UB: Uniform Billing
US: United States
